# Protective effect of Glycyrrhizin on cuprizone-induced model of multiple sclerosis in male C57BL/6 mice

**DOI:** 10.1016/j.ibneur.2026.07.012

**Published:** 2026-07-28

**Authors:** Asal sadat Morakkabi, Shahin Hassanpour, Razieh Hosseini

**Affiliations:** Department of Basic Veterinary Sciences, SR.C., Islamic Azad University, Tehran, Iran

**Keywords:** Cuprizone, Glycyrrhizin, Multiple sclerosis, Mice

## Abstract

This research focused on assessing the protective effect of glycyrrhizin in male C57BL/6 mice with cuprizone-induced (CPZ) multiple sclerosis (MS). Forty male C57BL/6 mice were divided into four experimental groups. The control group was given the regular diet. In group 2, mice were administered a diet that included 0.2% (w/w) CPZ blended with ground chow for 4 weeks (Zhu et al., 2021). In group 3, mice were given glycyrrhizin (400 mg/kg) by mouth daily for 4 weeks. In group 4, mice were given a diet containing 0.2% CPZ and glycyrrhizin (400 mg/kg) by mouth for 4 weeks. Following the occurrence of the MS sign, reflexive motor behavior and levels of serum antioxidants were evaluated. According to the results, the administration of CPZ notably reduced rotarod duration, frequency of crossings in the open field test (OFT), hindlimb foot angle, suspension of front and hind limbs, as well as grip strength (P < 0.05). The administration of CPZ notably raised serum malondialdehyde (MDA), tumor necrosis factor (TNF-α), and interleukin-1 beta (IL-1β), while simultaneously lowering superoxide dismutase (SOD), glutathione peroxidase (GPX), and catalase (TAS) levels (P < 0.05). Glycyrrhizin notably enhanced stay duration on rotarod, quantity of crossings in OFT, hindlimb foot angle, front- and hindlimb suspension, and grip strength (P < 0.05). Glycyrrhizin notably lowered serum MDA, TNF-α, and IL-1β, while elevating SOD, GPX, and TAS levels (P < 0.05). These findings indicated that glycyrrhizin offers a protective effect against CPZ-induced multiple sclerosis in mice.

## Introduction

The licorice (liquorice) plant boasts a long history of utilization in both Eastern and Western cultures. The genus name Glycyrrhiza is derived from the ancient Greek phrase meaning 'sweet root'. Glycyrrhizin, present in licorice root, is an essential element that has been utilized for ages in traditional medicine and is recognized for its significant levels of Glycyrrhizin, which contribute to its diverse effects (Nazari et al. 2017). Glycyrrhizin is made up of a single glycyrrhetinic acid molecule attached to two glucuronic acid molecules. From a structural perspective, Glycyrrhizin, a form of triterpene saponin, is frequently incorporated into food and cosmetic items as a flavoring agent. It has also been proposed for possible medical applications because of its various health benefits. Glycyrrhizin exhibits pharmacological and biological effects that include antiviral, anti-inflammatory, antioxidant, and anticancer properties (Bakr et al. 2022).

Multiple sclerosis (MS) is a long-term inflammatory and neurodegenerative disorder of the central nervous system (CNS), marked by the loss of myelin on nerve fibers, the deterioration of neural structures, and the loss of axons (Mey et al. 2023). The primary characteristics of MS include motor impairment, sensory issues, and cognitive challenges. Cognitive impairment represents a critical aspect of MS, impacting around 43–70% of those affected (Gómez-Melero et al. 2024). The cognitive areas most significantly impacted by MS are processing speed and working memory. While the precise pathophysiological mechanisms contributing to cognitive deficits in MS are not fully understood, but white matter lesions, grey matter atrophy, and changes in connectivity among grey matter areas like the hippocampus and cerebral cortex (Benedict et al. 2020).

Cuprizone (CPZ) serves as a copper chelator that is frequently employed to reliably induce demyelination in rodent models. Exposure to CPZ disrupts the energy metabolism within oligodendrocytes, consequently leading to their programmed cell death and instigating neuroinflammatory processes in the cerebral environment by activating microglia and astrocytes (Zirngibl et al. 2022). Moreover, an abundance of behavioral changes can be noted in mice administered with CPZ, which include alterations in motor skills, signs of pain-like symptoms, along with deficits in mental and cognitive functions (Aldhahri et al. 2022).

Among the different animal models used to investigate MS, the CPZ model has been recognized as especially beneficial for examining cognitive impairments linked to MS. The demyelination pattern noted in the hippocampus and prefrontal cortex in the CPZ model aligns with the known patterns and physiological traits linked to MS (Mooshekhian et al. 2022). Based on reports, phytoconstituents in Licorice *(Glycyrrhiza glabra L.)* has antioxidant and anti-inflammatory activites that could enhance histological dermal and epidermal properties, and reduce the level of inflammatory and wrinkling markers (Fatoki et al. 2023). Also, glycyrrhizin has anti-inflammatory activity which reduced inflammatory cell infiltration and demyelination, decreased tumor necrosis factor-alpha (TNF-α), IFN-γ, IL-17A, IL-6 and increased IL-4 both in serum and spinal cord homogenate as well as astrocytes and microglia. In a similar study it is reported, 18 β-Glycyrrhetinic acid alleviates demyelination by modulating the microglial phenotype in CPZ-induced demyelination (Tian et al. 2021). However, there is no report regarding protective effect of glycyrrhizin in reflexicve motor behavior on CPZ-induced model of MS in male mice. Thus, this paper aimed to determine protective effect of glycyrrhizin maniely in reflexicve motor behavior and subsequently serum antioxidant and anti-inflamatory biomarkers on CPZ-induced model of MS in male mice.

## Material and methods

### Animals

Forty male C57BL/6 mice, aged between 4 and 6 weeks and exhibiting a weight of 20 ± 2 g, were maintained under controlled environmental conditions (temperature: 22°C ± 2°C, with a 12-hour light/dark cycle). The subjects had continuous access to standard food and water. After a one-week acclimatization period, the mice were randomly allocated into four distinct groups (n = 10). The ethical research committee of Islamic Azad University, Science and Research Branch, granted approval for the study protocols. Subsequently, the animals were assigned to four experimental groups. The control group was administered a standard diet. In the second group, mice were provided with a diet comprising 0.2% (w/w) cuprizone (CPZ) (Sigma Aldrich, St. Louis, MO, USA) blended with ground chow for a duration of four weeks (Zhu et al. 2021). In the third group, mice received glycyrrhizin (Sigma Aldrich, St. Louis, MO, USA, at a dosage of 400 mg/kg) orally on a daily basis for a period of four weeks. In the fourth group, the mice were given a diet containing 0.2% CPZ along with Glycyrrhizin (400 mg/kg) administered orally for four weeks (Lee et al., 2007, Zhou et al., 2015, Yang et al., 2019). The evaluation of disease severity was conducted utilizing the experimental autoimmune encephalomyelitis (EAE) scoring system, which quantifies severity on a scale from 0 to 5, delineated as follows: 0: absence of disease, 1: diminished tail tone, 2: weakness in the hind limbs, 3: paralysis in the hind limbs, 4: paralysis or weakness in both hind limbs and forelimbs, and 5: moribund state or death (Graves and Montalban, 2020). Subsequently, reflexive motor behavior alongside serum antioxidant levels were evaluated.

## Behavioral tests

### Rotarod test

This test assessed motor coordination and balance. Mice were placed on a rotating rod, and the time until falling off was recorded.

### Open field test

The test was done using a 45 × 45 × 30 cm^3^ wooden box. The floor of the open field box was divided by masking tape markers into 9 squares. Each animal was placed individually at the center of the apparatus. Then, the number of segments crossed with the four paws was recorded for a period of 6 min (Donato et al. 2014).

### Front limb suspension

This test evaluated the ability of animals to hang by their front limbs. Briefly, mice grasped a wire suspended between two ends. The time until mice released the wire was recorded. This test was repeated three times, and the average time in seconds was recorded (Feather-Schussler and Ferguson, 2016).

### Hind limb suspension

Mice's hind legs were hung over a wire tied between two ends, and their posture was observed. Hind limb posture was scored based on the time to grasp the wire and the separation of hind limbs. The test was performed thrice to ensure consistency and account for learning effects (Feather-Schussler and Ferguson, 2016).

### Hind limb foot angle

Mouse movements were recorded in an open field box using a camera. Images capturing hind limb positions during full stride were used. A line was drawn from the heel to the tip of the toe, and the angle between them was measured (Feather-Schussler and Ferguson, 2016).

### Grip strength

Mice were placed on a fiberglass screen and slowly tilted from horizontal to vertical. Mice attempted to grip the screen to prevent falling. The time until falling was recorded. The test was performed thrice to ensure consistency and account for learning effects (Corti, 2017).

### Negative geotaxis

Mice were placed face down on a 45° incline. The time taken to turn and face upwards was recorded. The time until falling was recorded. The test was performed thrice to ensure consistency and account for learning effects (Feather-Schussler and Ferguson, 2016).

### Surface righting

The experiment consisted of putting mice in a supine posture on a cotton sheet, then measuring the time it took for them to revert to their prone position after being released for 5 s. The exam was administered three times in 3 min to ensure correctness since there was no evidence of a repeated learning effect (Feather-Schussler and Ferguson, 2016).

### Antioxidant activity

At the end of the study, animals were euthanized via an intraperitoneal overdose (50 mg/kg) of sodium thiopental (Rotexmedica, Germany; following AVMA Guidelines for the Euthanasia of Animals 'No: S5.2.1.1′, Acceptable Methods; non-inhaled agents). Blood was taken from the cardiac immediately after the final behavioral test to measure serum MDA, SOD, GPx, CAT, TNF-α and IL-1β activities using Randox (UK, England) ELISA assay kits.

### Statistical analysis

Data were analyzed using one-way ANOVA and were presented as mean ± SE (standard error) using SPSS version 22.0. For treatments showing significant differences by ANOVA, between-group evaluations were performed using the Tukey posthoc test (p < 0.05).

## Results

**Fig. 1:**
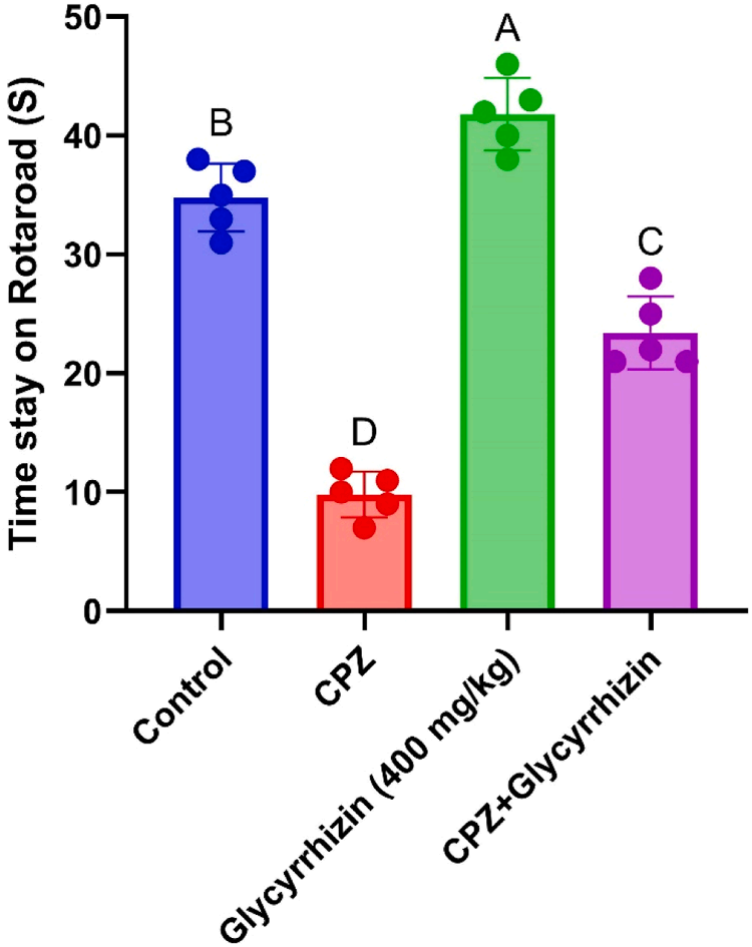
Effects of the Glycyrrhizin on time stay on rotarod test in experimental cuprizone-induced Multiple Sclerosis Male C57BL/6 mice. Data are expressed as mean ± SE. There are significant differences between groups with different superscripts (a-d; P < 0.05).

As seen in Fig. 1, CPZ significantly decreased time satay on rotarod compared to control group (P < 0.05). Glycyrrhizin significantly increased stay on rotarod than to control group (P < 0.05). Co-administration of the Glycyrrhizin and CPZ significantly decreased adverse effects of the CPZ compared to the CPZ group (P < 0.05).

**Fig. 2:**
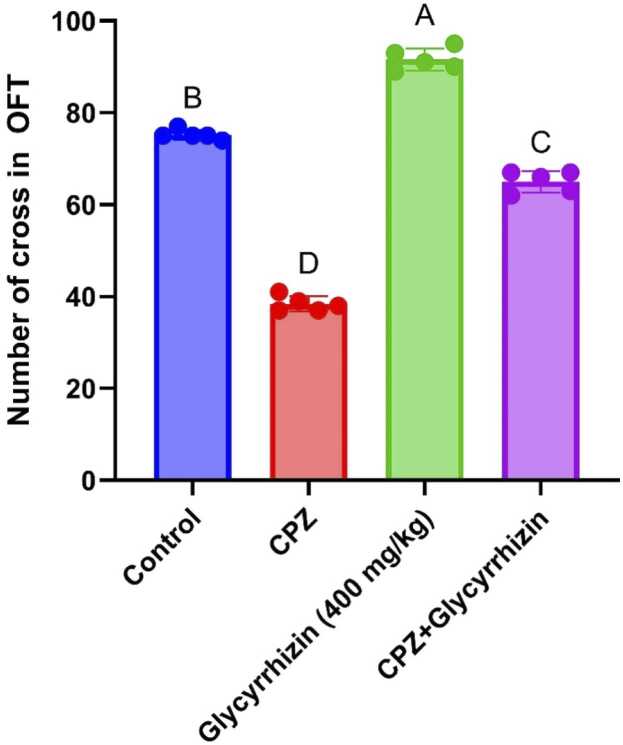
Effects of the Glycyrrhizin on number of cross in open field tests (OFT) in experimental cuprizone-induced Multiple Sclerosis Male C57BL/6 mice. Data are expressed as mean ± SE. There are significant differences between groups with different superscripts (a-d; P < 0.05).

Based on Fig. 2, CPZ significantly decreased number of cross in OFT on rotarod compared to control group (P < 0.05). Glycyrrhizin significantly increased number of cross in OFT than to control group (P < 0.05). Co-administration of the Glycyrrhizin and CPZ significantly decreased adverse effects of the CPZ compared to the CPZ group (P < 0.05).

**Fig. 3:**
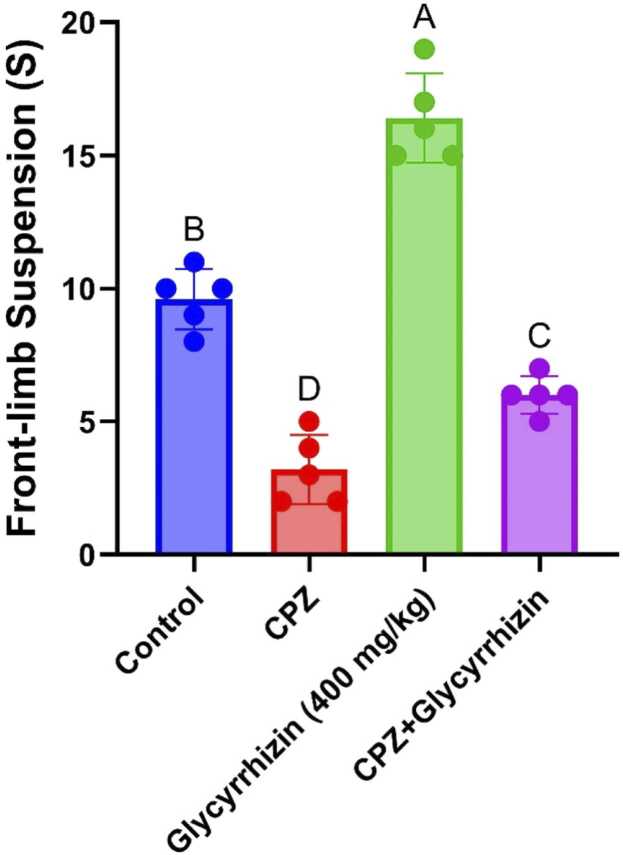
Effects of the Glycyrrhizin on front-limb suspension in experimental cuprizone-induced Multiple Sclerosis Male C57BL/6 mice. Data are expressed as mean ± SE. There are significant differences between groups with different superscripts (a-d; P < 0.05).

In Fig. 3, CPZ significantly decreased front-limb suspension compared to control group (P < 0.05). Glycyrrhizin significantly increased front-limb suspension than to control group (P < 0.05). Co-administration of the Glycyrrhizin and CPZ significantly decreased adverse effects of the CPZ compared to the CPZ group (P < 0.05).

**Fig. 4:**
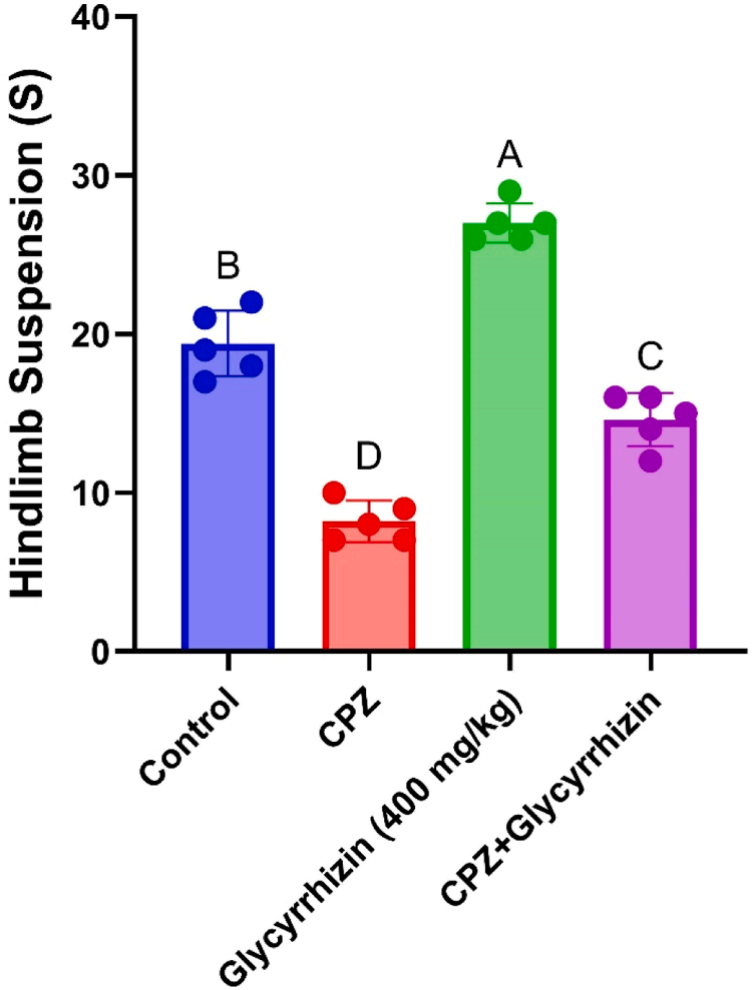
Effects of the Glycyrrhizin on hind-limb suspension in experimental cuprizone-induced Multiple Sclerosis Male C57BL/6 mice. Data are expressed as mean ± SE. There are significant differences between groups with different superscripts (a-d; P < 0.05).

As shown in Fig. 4, CPZ significantly decreased hind-limb suspension compared to control group (P < 0.05). Glycyrrhizin significantly increased hind-limb suspension than to control group (P < 0.05). Co-administration of the Glycyrrhizin and CPZ significantly decreased adverse effects of the CPZ compared to the CPZ group (P < 0.05).

**Fig. 5:**
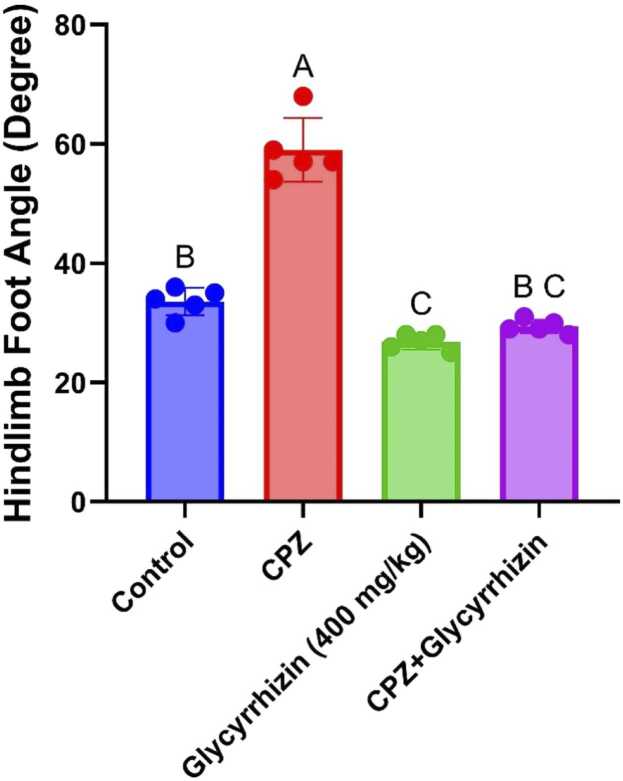
Effects of the Glycyrrhizin on hind-limb foot angel in experimental cuprizone-induced Multiple Sclerosis Male C57BL/6 mice. Data are expressed as mean ± SE. There are significant differences between groups with different superscripts (a-c; P < 0.05).

According to Fig. 5, CPZ significantly increased hind-limb foot angel compared to control group (P < 0.05). Glycyrrhizin significantly decreased hind-limb foot angel than to control group (P < 0.05). Co-administration of the Glycyrrhizin and CPZ had no significant effect on adverse effects of the CPZ compared to the CPZ group (P < 0.05).

**Fig. 6:**
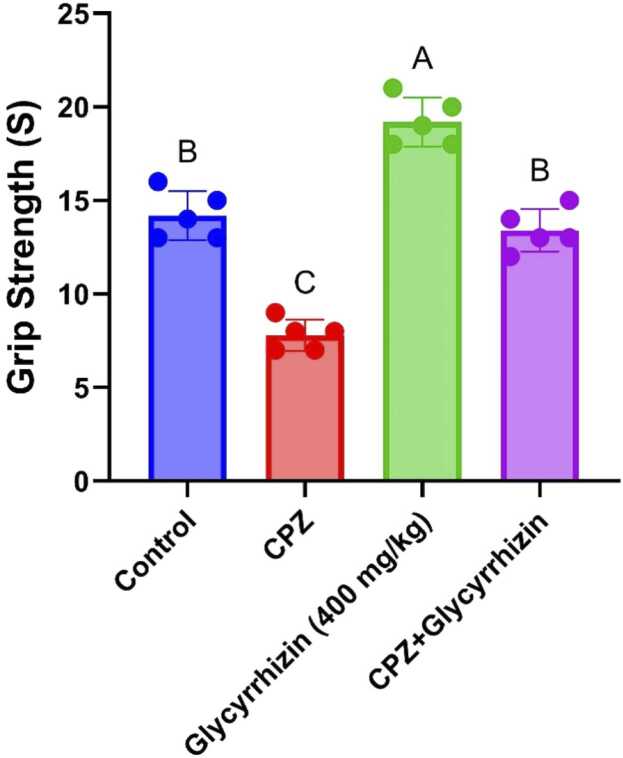
Effects of the Glycyrrhizin on grip strength in experimental cuprizone-induced Multiple Sclerosis Male C57BL/6 mice. Data are expressed as mean ± SE. There are significant differences between groups with different superscripts (a-c; P < 0.05).

Due to Fig. 6, CPZ significantly decreased on grip strength compared to control group (P < 0.05). Glycyrrhizin significantly increased on grip strength than to control group (P < 0.05). Co-administration of the Glycyrrhizin and CPZ significantly decreased adverse effects of the CPZ compared to the CPZ group (P < 0.05).

**Fig. 7:**
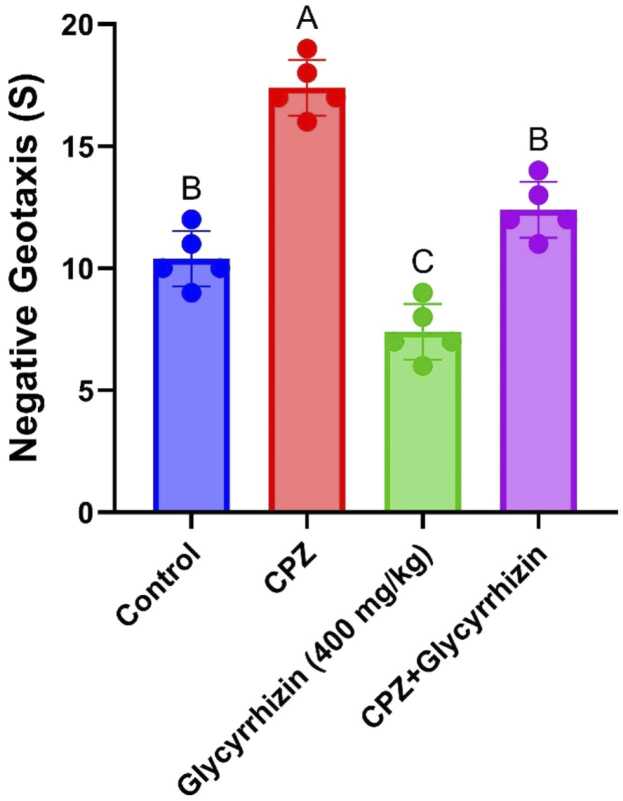
Effects of the Glycyrrhizin on negative geotaxis in experimental cuprizone-induced Multiple Sclerosis Male C57BL/6 mice. Data are expressed as mean ± SE. There are significant differences between groups with different superscripts (a-c; P < 0.05).

In this study, CPZ significantly increased on negative geotaxis compared to control group (P < 0.05). Glycyrrhizin significantly decreased negative geotaxis than to control group (P < 0.05). Co-administration of the Glycyrrhizin and CPZ significantly decreased adverse effects of the CPZ compared to the CPZ group (P < 0.05) (Fig. 7).

**Fig. 8:**
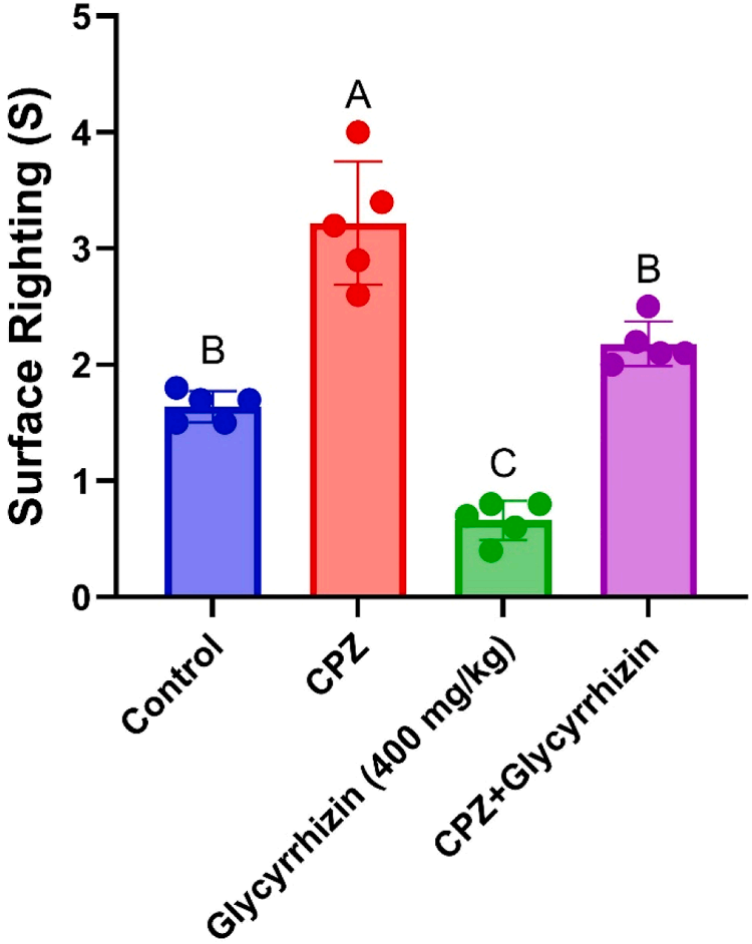
Effects of the Glycyrrhizin on surface righting in experimental cuprizone-induced Multiple Sclerosis Male C57BL/6 mice. Data are expressed as mean ± SE. There are significant differences between groups with different superscripts (a-c; P < 0.05).

In shown, CPZ significantly increased on surface righting compared to control group (P < 0.05). Glycyrrhizin significantly decreased surface righting than to control group (P < 0.05). Co-administration of the Glycyrrhizin and CPZ significantly decreased adverse effects of the CPZ compared to the CPZ group (P < 0.05) (Fig. 8).

**Fig. 9:**
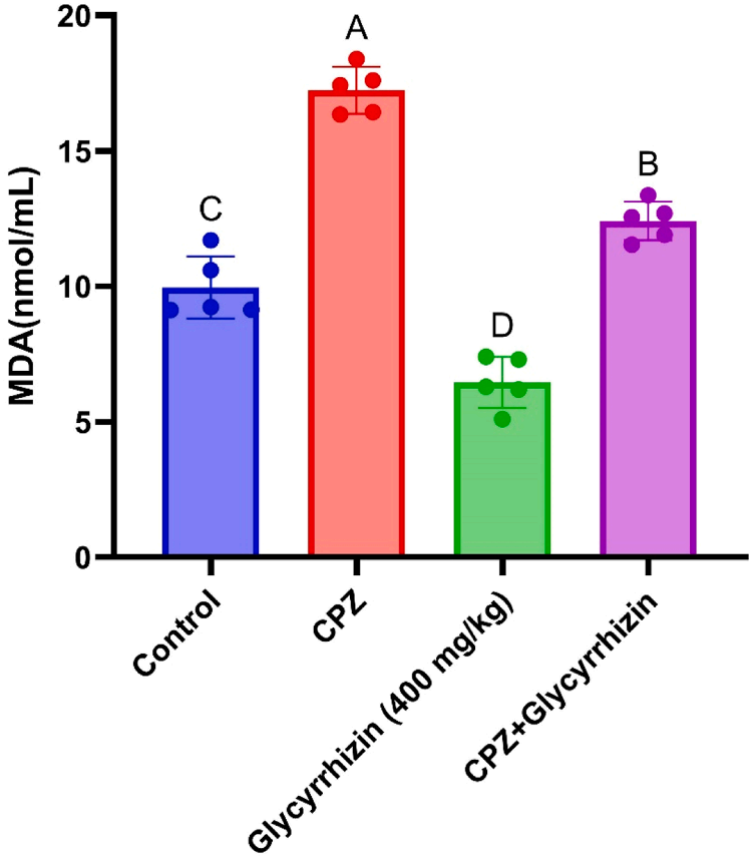
Effects of the Glycyrrhizin on serum malondialdehyde (MDA) in experimental cuprizone-induced Multiple Sclerosis Male C57BL/6 mice. Data are expressed as mean ± SE. There are significant differences between groups with different superscripts (a-d; P < 0.05).

Based on Fig. 9, CPZ significantly increased serum MDA levels compared to control group (P < 0.05). Glycyrrhizin significantly decreased serum MDA levels than to control group (P < 0.05). Co-administration of the Glycyrrhizin and CPZ significantly decreased adverse effects of the CPZ compared to the CPZ group (P < 0.05).

**Fig. 10:**
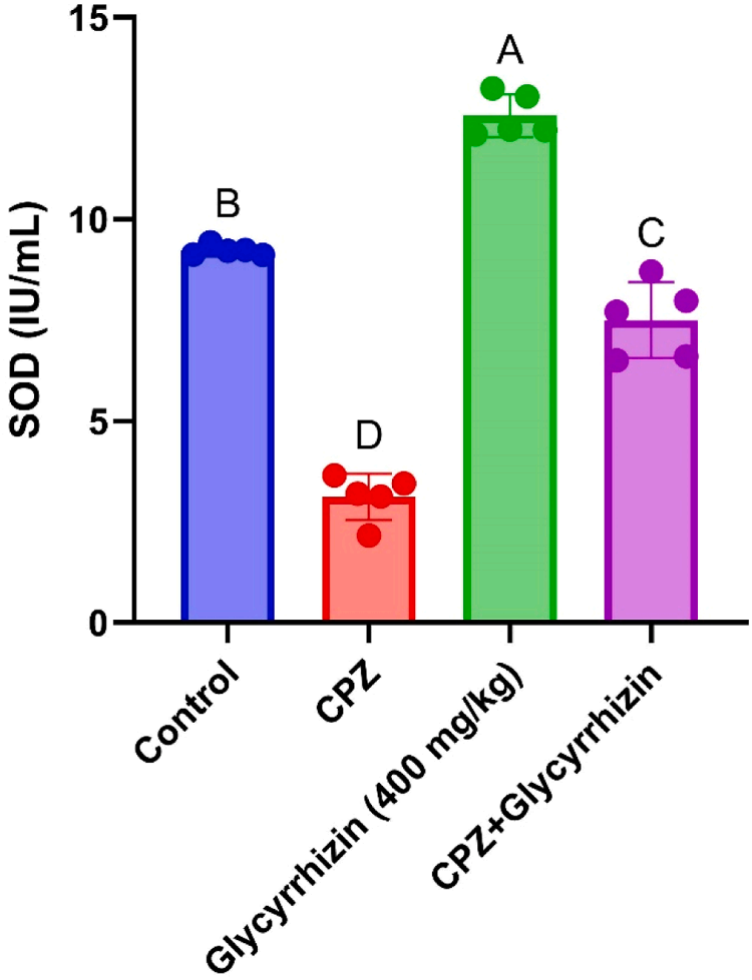
Effects of the Glycyrrhizin on serum superoxide dismutase (SOD) in experimental cuprizone-induced Multiple Sclerosis Male C57BL/6 mice. Data are expressed as mean ± SE. There are significant differences between groups with different superscripts (a-d; P < 0.05).

Also, based on Fig. 10, CPZ significantly decreased serum SOD levels compared to control group (P < 0.05). Glycyrrhizin significantly increased serum SOD levels than to control group (P < 0.05). Co-administration of the Glycyrrhizin and CPZ significantly decreased adverse effects of the CPZ compared to the CPZ group (P < 0.05).

**Fig. 11:**
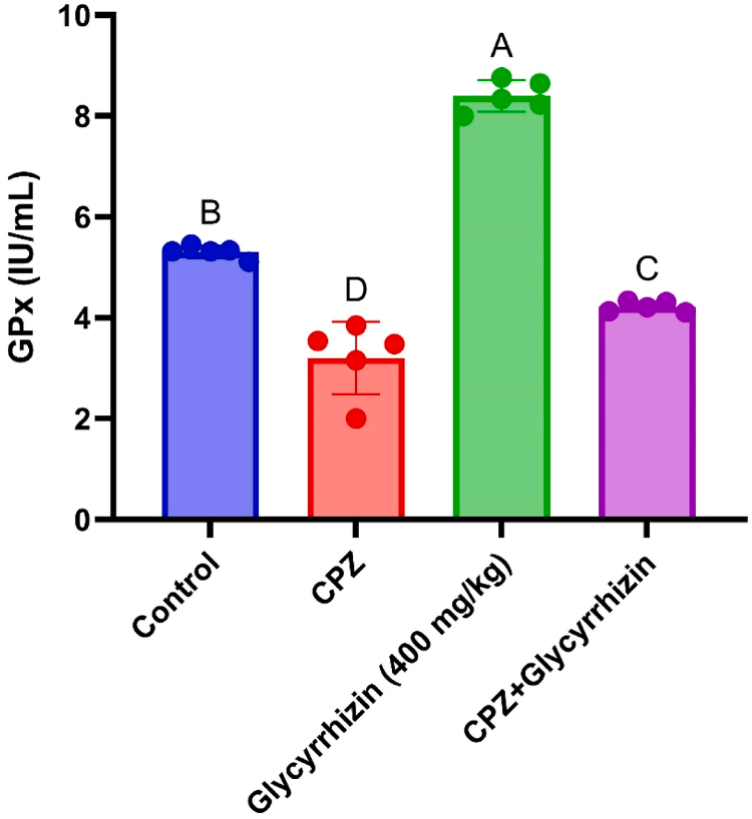
Effects of the Glycyrrhizin on serum glutathione peroxidase (GPx) in experimental cuprizone-induced Multiple Sclerosis Male C57BL/6 mice. Data are expressed as mean ± SE. There are significant differences between groups with different superscripts (a-d; P < 0.05).

Also, based on Fig. 11, CPZ significantly decreased serum GPx levels compared to control group (P < 0.05). Glycyrrhizin significantly increased serum GPx levels than to control group (P < 0.05). Co-administration of the Glycyrrhizin and CPZ significantly decreased adverse effects of the CPZ compared to the CPZ group (P < 0.05).

**Fig. 12:**
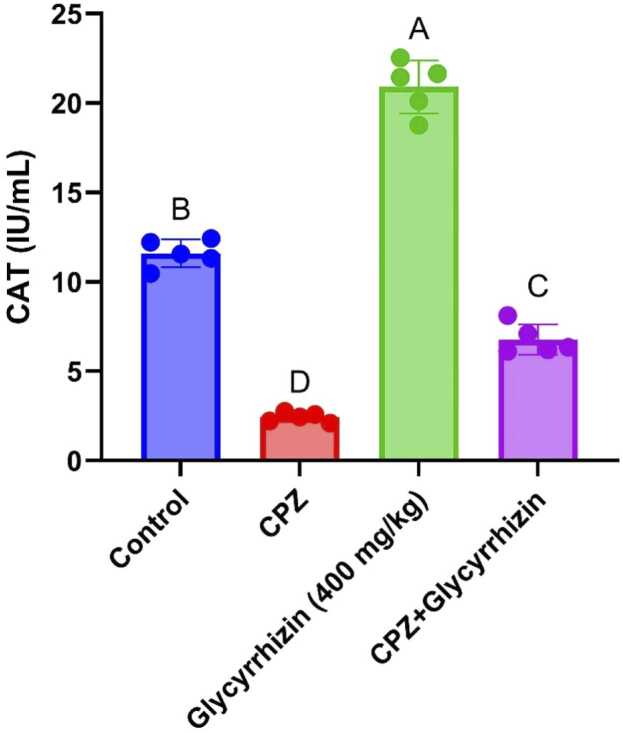
Effects of the Glycyrrhizin on serum catalase (CAT) in experimental cuprizone-induced Multiple Sclerosis Male C57BL/6 mice. Data are expressed as mean ± SE. There are significant differences between groups with different superscripts (a-d; P < 0.05).

Additionally, CPZ significantly decreased serum CAT levels compared to control group (P < 0.05). Glycyrrhizin significantly increased serum CAT levels than to control group (P < 0.05). Co-administration of the Glycyrrhizin and CPZ significantly decreased adverse effects of the CPZ compared to the CPZ group (P < 0.05) (Fig. 12).

**Fig. 13:**
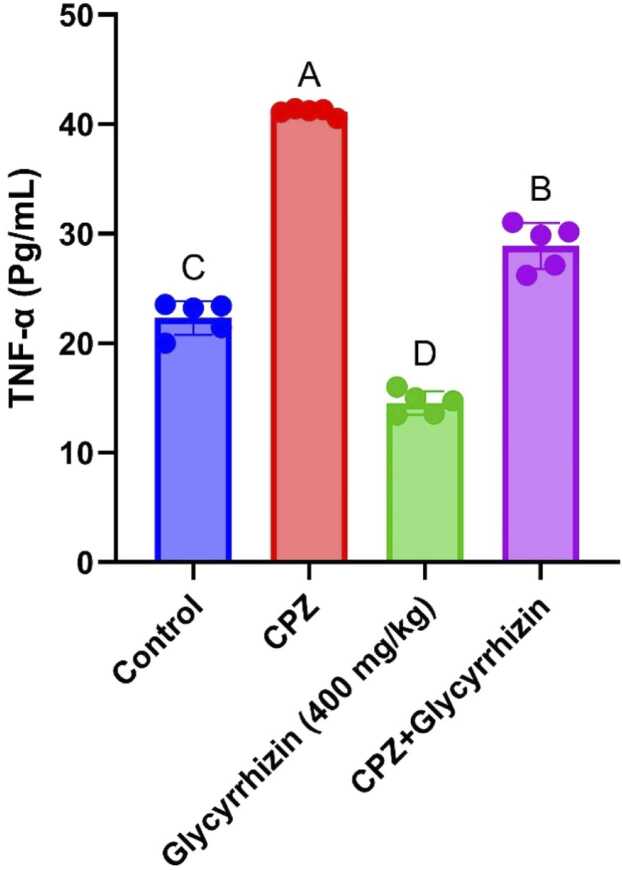
Effects of the Glycyrrhizin on serum tumor necrosis alpha (TNF-α) in experimental cuprizone-induced Multiple Sclerosis Male C57BL/6 mice. Data are expressed as mean ± SE. There are significant differences between groups with different superscripts (a-d; P < 0.05).

As seen, in Fig. 13, CPZ significantly increased serum TNF-α levels compared to control group (P < 0.05). Glycyrrhizin significantly decreased serum TNF-α levels than to control group (P < 0.05). Co-administration of the Glycyrrhizin and CPZ significantly decreased adverse effects of the CPZ compared to the CPZ group (P < 0.05).

**Fig. 14:**
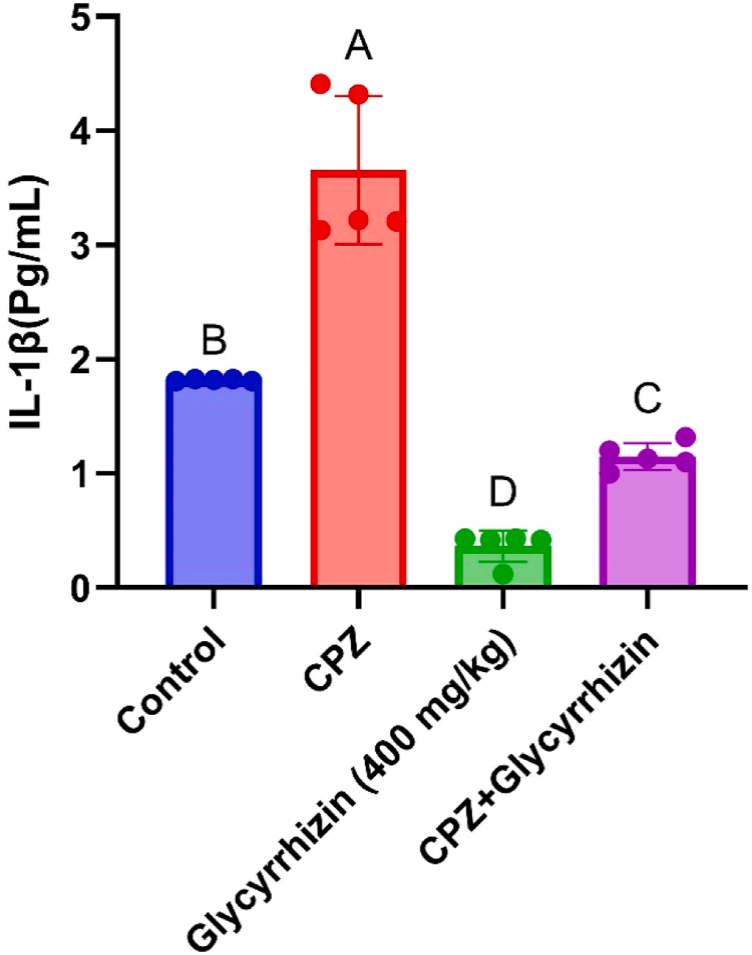
Effects of the Glycyrrhizin on serum interleukin 1-beta (IL-1β) in experimental cuprizone-induced Multiple Sclerosis Male C57BL/6 mice. Data are expressed as mean ± SE. There are significant differences between groups with different superscripts (a-d; P < 0.05).

According to Fig. 14, CPZ significantly increased serum IL-1β levels compared to control group (P < 0.05). Glycyrrhizin significantly decreased serum IL-1β levels than to control group (P < 0.05). Co-administration of the Glycyrrhizin and CPZ significantly decreased adverse effects of the CPZ compared to the CPZ group (P < 0.05).

## Discussion

Based on main findings of the current study, administration of CPZ notably reduced rotarod duration and reflexive motor behavior indexes in mice. Also, administration of CPZ notably raised serum MDA, TNF-α, and interleukin−1 beta (IL-1β), while simultaneously lowering SOD, GPX, and TAS levels. Numerous methodologies have been proposed for a demyelination model wherein a diet enriched with CPZ over a duration of 4–6 weeks is favored by a significant contingent of researchers (Li et al. 2022). This phenomenon culminates in the impairment of oligodendrocytes, subsequently triggering the activation of microglia and astrocytes, thereby perturbing mitochondrial energy metabolism. Consequently, the use of C57BL/6 mice has been advocated and in the present investigation, we employed a CPZ-containing diet for a duration of 4 weeks utilizing C57BL/6 mice (Xu et al. 2009). Our findings indicate that CPZ markedly diminished the frequency of crossings and the performance on the rotarod test, while concomitant administration of CPZ with CEO mitigated the detrimental effects associated with CPZ. In this context, Xu et al. [14] reported that mice subjected to a dosage of 0.2% CPZ for a period of 4–6 weeks exhibited compromised sensorimotor gating and diminished social engagement, leading to prolonged occupancy in the open arms of the maze and exacerbated memory deficits. Demyelination predominantly transpires within white matter; however, there exists corroborative evidence pertaining to alterations in grey matter (Zhu et al. 2021). The evidence suggests a strong link between the pathophysiology of MS and oxidative stress in both humans and animals. Due to elevated oxygen usage and reactive oxygen species generation, brain tissue is particularly vulnerable to oxidative harm caused by polyunsaturated fatty acids in neuronal membranes. Consequently, excess MDA production and diminished intracellular antioxidant defenses (such as catalase, GPx, and SOD) result in dysfunction and eventually cause neuronal cell death (Ramos-González et al. 2024).

According to the results, the administration of the glycyrrhizin notably enhanced stay duration on rotarod, reflexive motor behavior indexes in mice. Glycyrrhizin notably lowered serum MDA, TNF-α, and IL-1β, while elevating SOD, GPX, and TAS levels. Neurodevelopmental reflexes are involuntary and repetitive movements. This illustrates the reflexes of the brain stem and spinal cord that are observed in newborns and infants. Changes in brain development can result in abnormal synaptogenesis, functioning, and myelination, which may lead to delays or absences in neurodevelopmental reflexes (Nguyen et al. 2017). The array of reflexes described includes, but is not limited to, limb grasping and placing, cliff avoidance, righting, accelerated righting, gait, auditory startle, posture, and eye opening (Nguyen et al. 2017). Abnormal reflexes can result in delays in acquisition, absence, or reappearance throughout life. Therefore, experimental models of neurodevelopmental disabilities can be utilized to assess reflex delays, with rodents serving as the optimal animal model to replicate these motor skills (Semple et al. 2013). CPZ addministration leads to EAE and disturb motor skills.

In a study, it is reported 18β-glycyrrhetinic acid modulated microglia suppresses EAE through inhibiting microglia activation-mediated CNS inflammation, and promoting neuroprotective effect of microglia, which represents a potential therapeutic strategy for MS and maybe other neuroinflammatory diseases associated with microglia activation (Zhou et al. 2015). Glycyrrhetinic acid exhibited a significant capacity to modulate the activation of microglia, resulting in a diminished pro-inflammatory profile within the CNS of EAE murine models (Zhou et al. 2015). Glycyrrhizin improved the recovery of neurological function, reduced lesion volume, and significantly decreased the expression of cyclooxygenase−2 (COX−2) and inducible nitric oxide synthase (iNOS) following glycyrrhizin treatment in the cerebral cortex and hippocampal dentate gyrus (Song et al. 2013).

This modification led to a decrease in the recruitment of encephalitogenic T lymphocytes into the CNS, thereby mitigating inflammation-induced demyelination within this region. Furthermore, microglia were found to be active during the induction phase of EAE, and the therapeutic efficacy of glycyrrhetinic acid is attributed to its capacity to inhibit microglial activation rather than affecting the encephalitogenic T cells (Kamisli et al. 2018). The expression levels of brain-derived neurotrophic factor (BDNF) exhibited a decrement correlating with the escalation of EAE severity. Conversely, in the cohort of mice administered with glycyrrhetinic acid, BDNF demonstrated a significant up-regulation within the CNS, with its expression pattern mirroring that of myelin basic protein and proteolipid protein. Glycyrrhizin inhibited microglial activation triggered by LPS, and glycyrrhizinate was observed to alleviate neuroinflammation induced by amyloid-beta (Aβ) in male mice (Guo et al. 2016) and our finding was in agrrement to this report.

The administration of glycyrrhizic acid (50 mg/kg/day, I.P.) has been demonstrated to decrease the expression levels of MDA, IL-1β, IL-6, and TNF-α, concurrently leading to a reduction in microglial activation (Ojha et al., 2016). Furthermore, the oral intake of glycyrrhizin (30 mg/kg) was found to mitigate postoperative neuroinflammation primarily by suppressing the synthesis of proinflammatory mediators (IL-1β, TNF-α, and IL-6) and inhibiting NF-κB nuclear expression in a model of surgery-induced cognitive impairment in aged C57Bl/6 mice (Kong et al. 2017) and our findings was in agreement to this report. Furthermore, glycyrrhetinic acid effectively mitigated the inhibition of BDNF expression induced by Interferon-gamma (IFN-γ) in primary microglial cultures in vitro. These findings suggest that the remyelination process observed in glycyrrhetinic acid-treated mice may be ascribed to the enhancement of BDNF expression facilitated by glycyrrhetinic acid-modulated microglial activities (Yang et al. 2019). However, based on limitations, in our study glycyrrhizin notably lowered serum MDA, TNF-α, and IL-1β but we were not able to etermine BDNF and IFN-γ levles. The administration of oral 18β-glycyrrhetinic acid led to a significant increase in the expression of myelin basic protein (MBP) when compared to the CPZ-toxic mice. Furthermore, treatment with 18β-glycyrrhetinic acid has been shown to effectively diminish the demyelination caused by CPZ (Tian et al. 2021). Despite in the current study we were not able to detemrine MBP levles, but similar results obtained on protective effect of the glycyrrhizin on CPZ-induced MS in mice.

Glycyrrhizin has been effectively utilized in the treatment of various autoimmune disorders, such as arthritis, autoimmune thyroid disease, and autoimmune hepatitis. In the experimental model of EAE using C57BL/6 mice, glycyrrhizin treatment (10, 25, and 50 mg/kg, I.P.) demonstrated the ability to inhibit reactive glial cells, safeguard against neuronal damage, and provide a protective effect against clinical symptoms, inflammation, and demyelination (Paudel et al. 2020). Perhaps via this mevhanisms glycyrrhizin improved reflexive motor behavior in CPZ-treated male C57BL/6 mice. In conclusion, these findings indicated that glycyrrhizin offers a protective effect against CPZ-induced MS in mice. Due to constraints, we could not establish histological or molecular analyses for the results obtained. Additional studies involving histological and molecular analyses indicated.

## CRediT authorship contribution statement

**Razieh Hosseini:** Project administration. **Asal Sadat Morakkabi:** Writing – original draft, Data curation. **Shahin Hassanpour:** Supervision, Methodology.

## Declaration of Competing Interest

No conflict of interest.

## Data Availability

No data was used for the research described in the article.
